# The Comparison of the Effects of Three Physiotherapy Techniques on Hamstring Flexibility in Children: A Prospective, Randomized, Single-Blind Study

**DOI:** 10.1371/journal.pone.0072026

**Published:** 2013-08-12

**Authors:** Dariusz Czaprowski, Justyna Leszczewska, Aleksandra Kolwicz, Paulina Pawłowska, Agnieszka Kędra, Piotr Janusz, Tomasz Kotwicki

**Affiliations:** 1 Department of Physiotherapy, Józef Rusiecki University College, Olsztyn, Poland; 2 Rehasport Clinic, Poznań, Poland; 3 Faculty of Physical Education and Sport in Biała Podlaska, Józef Piłsudski University of Physical Education, Warsaw, Poland; 4 Department of Pediatric Orthopedics and Traumatology, University of Medical Sciences, Poznań, Poland; University of South Australia, Australia

## Abstract

The aim of the study was to evaluate changes in hamstring flexibility in 120 asymptomatic children who participated in a 6-week program consisting of one physiotherapy session per week and daily home exercises. The recruitment criteria included age (10–13 years), no pain, injury or musculoskeletal disorder throughout the previous year, physical activity limited to school sport. Subjects were randomly assigned to one of the three groups: (1) post-isometric relaxation – PIR (n = 40), (2) static stretch combined with stabilizing exercises – SS (n = 40) and (3) stabilizing exercises – SE (n = 40). Hamstring flexibility was assessed with straight leg raise (SLR), popliteal angle (PA) and finger-to-floor (FTF) tests. The examinations were conducted by blinded observers twice, prior to the program and a week after the last session with the physiotherapist. Twenty-six children who did not participate in all six exercise sessions with physiotherapists were excluded from the analysis. The results obtained by 94 children were analyzed (PIR, n = 32; SS, n = 31; SE, n = 31). In the PIR and SS groups, a significant (P<0.01) increase in SLR, PA, FTF results was observed. In the SE group, a significant (P<0.001) increase was observed in the SLR but not in the PA and FTF (P>0.05). SLR result in the PIR and SS groups was significantly (P<0.001) higher than in the SE group. As far as PA results are concerned, a significant difference was observed only between the SS and SE groups (P = 0.014). There were no significant (P = 0.15) differences regarding FTF results between the three groups. Post-isometric muscle relaxation and static stretch with stabilizing exercises led to a similar increase in hamstring flexibility and trunk forward bend in healthy 10–13-year-old children. The exercises limited to straightening gluteus maximus improved the SLR result, but did not change the PA and FTF results.

## Introduction

Reduced hamstring flexibility is a common clinical finding in adolescents [1], [2], [3]. Brodersen et al. observed that 75% of boys and 35% of girls aged 10 revealed reduced flexibility of hamstrings [1]. Harreby et al. confirmed this observation in 15- to 17-year-old boys [2]. Reduced flexibility of hamstrings was reported to be associated with increased low-back pain prevalence [4]–[8], herniated lumbar disc [8], [9], decreased lumbar lordosis [5], decreased range of lumbar spine flexion and increased range of thoracic spine flexion [10], increased thoracic kyphosis angle in adolescents with Scheuermann disease [11] and a higher risk of muscle injury [12].

Therefore, various stretching techniques are used in clinical practice to increase hamstring flexibility [13]–[20]. One of them is post-isometric relaxation (PIR) [21], [22], a muscle energy technique (MET) which refers to reduced muscle tonus experienced in a brief period following its isometric contraction [21], [22]. Post-isometric relaxation is considered to be an effective method of increasing hamstring flexibility [23]–[25].

Another technique is static stretching (SS), which focuses on maintaining the end-range position of the joint with simultaneous slight stretch in the trained muscles [16], [17], [26], [27]. It is recommended that static stretching should be supplemented with the activation of a muscle responsible for stabilizing the musculoskeletal system (e.g. gluteus maximus) [27], [28]. According to Mottram and Comerford [27] as well as Sahrmann [28], such a combination is efficient in shaping a correct pattern of muscular activity. The aim of this technique is to inhibit hyperactive muscles by using nervous system reaction to elongate the muscles and to activate inhibited stabilizing muscles [27], [28].

During rehabilitation programs as well as physical education lessons various exercises affecting gluteus maximus are applied [27]–[29]. The main aim of these exercises is to improve gluteus maximus strength [29]. However, Wagner et al. [30] observed that a rehabilitation program focusing on improving strength and neuromuscular control of the gluteus maximus resulted in a decrease in hamstring activation and led to improving its flexibility. Sewall and Micheli [31] noted a slight increase in hip joint range of flexion in prepubertal children participating in a progressive resistive strength training. Thus, it is interesting to evaluate if the exercises aimed at increasing the activity of gluteus maximus are able to change flexibility of hamstrings in children.

Several tests may be used to assess hamstring flexibility [15], [19], [21], [32]–[34], both in epidemiologic studies [2], [4]–[6] and in the verification of the effectiveness of stretching programs [14], [16], [19], [35]. Two most commonly used tests are the straight leg raise test (SLR) [15]–[16], [32] and the popliteal angle test (PA) [14], [33]–[36], both considered to be objective and reliable [32]–[34]. The evaluation of hamstring flexibility may be supplemented with the finger-to-floor test which, apart from hamstrings, assesses spine and gastrocnemius muscle flexibility [15], [37].

The aim of the study was to compare the effects of three physiotherapy techniques based on different mechanisms of influencing the musculoskeletal system: (1) post-isometric relaxation - PIR, (2) static stretch combined with stabilizing exercises - SS and (3) stabilizing exercises only – SE, on hamstring flexibility following a 6-week exercise program.

## Methods

### Trial design [38]

This was a single-center, prospective, randomized (allocation ratio 1∶1∶1), single-blind, parallel-group study conducted in Olsztyn, Poland.

### Subjects

The subjects were selected during 3 presentations for parents and their children. The presentations were organized in a randomly selected primary school located in Olsztyn, Poland. The school was selected by means of simple randomization with the use of a computer-generated number of the school.

Information concerning the dates of the meetings was published (with the school director's consent) on the school notice board and school website one month prior to the presentations. The meetings were open to all parents and children from grades 4, 5 and 6. At the time of the research these groups included 227 children in total.

**Figure 1:**
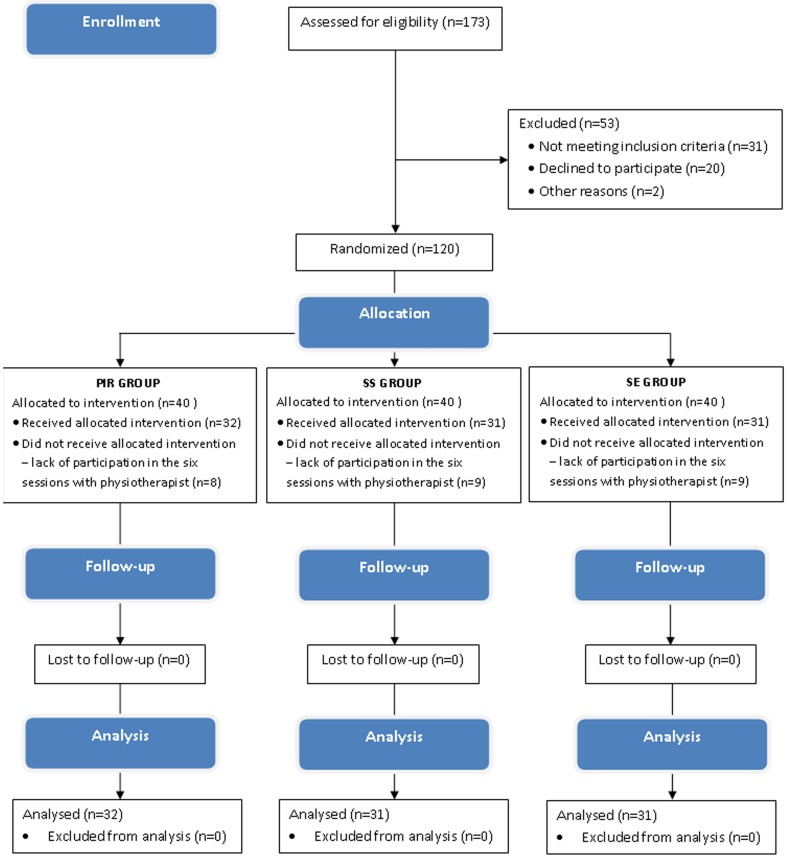
The protocol of the study.

173 parents with children participated in the meetings during which they were informed about the aim of the study, its schedule, recruitment criteria and the possibility to resign from the participation at any time. Finally, the study included 120 children who met the following recruitment criteria: (1) age 10–13 years, (2) no pain, injury or other musculoskeletal disorders throughout the previous year and (3) regular physical activity limited to school sport. The basic demographic parameters of the study group were as follows: age - 10–13 years, mean 11.5±0.5; height (m) 1.34–1.73, mean 1.53±0.07; weight (kg) 29.0–72.0, mean 44.4±10.1; BMI (kg m-2) 13.2–28.2, mean 18.9±3.5. All children attended the same school and participated in the same school sports program. The protocol of the study is presented in the CONSORT flow diagram [38] (Figure 1).

Prior to the study, a written parents' consent was obtained. The Józef Rusiecki University College Ethical Commission approved the study.

All the subjects of the photographs or their legal guardians have given written informed consent, as outlined in the PLOS consent form, to the publication of their photographs.

### Study settings

The study took place at the Center of Body Posture of Józef Rusiecki University College in Olsztyn, Poland, from February 2012 to June 2012. Olsztyn is the major city of Warmia and Mazury region in the north-east of Poland with 200 000 inhabitants.

### Measurement protocol

During the examinations the subjects were barefoot and wearing loose clothes. The examination was carried out twice: (1) the initial examination (Exam1) immediately prior to the exercise program and (2) the final examination (Exam2) one week after the last session with the physiotherapist; that day corresponded to the first day after the last session of home exercises. The examination was performed simultaneously by two observers. The observers were physiotherapists who did not participate in the randomization procedure and in the exercise program. It assured the study was single-blind. The first observer (O1) measured the angles with the use of inclinometer. The second observer (O2) did the test (SLR or PA) while controlling the subject's position visually and with palpation. The O2 controlled the following elements of the examination: (1) stable position of the pelvis, (2) lumbar spine flat on the table and (3) vertical position of the thigh during popliteal angle measurement. In order to assess hamstring flexibility, the SLR, the PA, and the FTF were applied. For the SLR and the PA, the right side results were considered. Measurements were made three times and the mean value was used in the analysis.

### Straight leg raise test (SLR) [15], [32]

**Figure 2:**
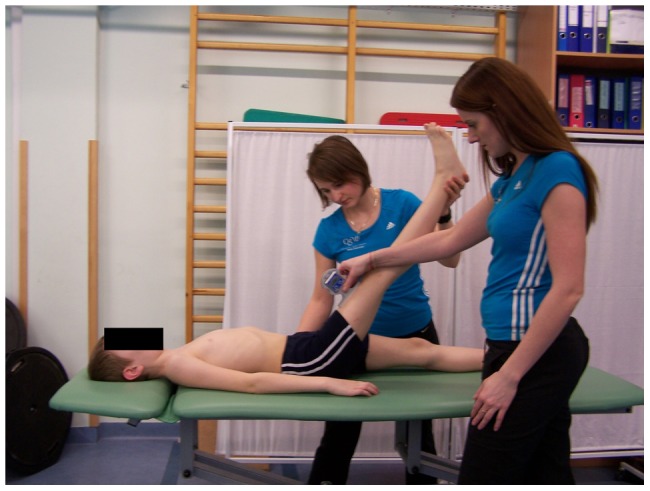
Straight leg raise test.

The test was carried out with the subject lying supine on a table with lower limbs extended and feet relaxed. The O2 controlled the position of the trunk and pelvis visually and with palpation, then raised the subject's right lower limb slowly to the point the subject felt resistance in hamstring muscles. Then O1 assessed the range of hip flexion using the AMI Digital Inclinometer (OPIW, Poland) (Figure 2). Before each measurement the inclinometer was reset in a horizontal position.

### Popliteal angle test (PA) [33], [34]

**Figure 3:**
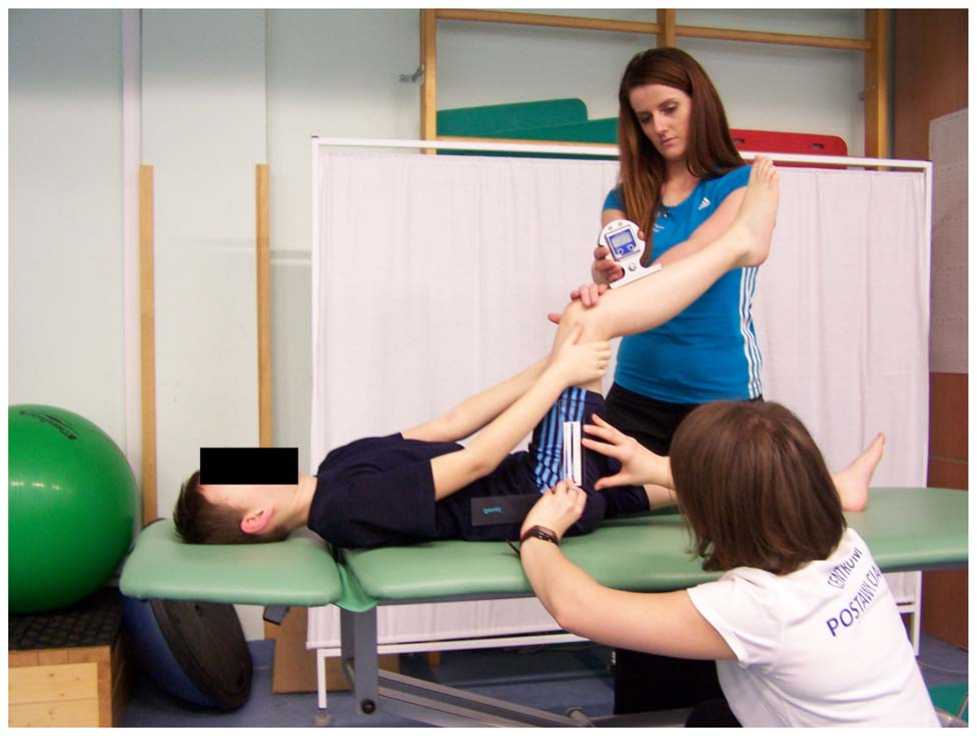
Popliteal angle test.

The popliteal angle was measured in a supine position with the right hip flexed to 90°. The child remained in this position with both hands placed on the posterior aspect of the right thigh. To control the 90° of hip flexion, the O2 used a goniometer, with the fulcrum set over the greater trochanter, the stationary arm parallel to the table, and the mobile arm along the femur. After assuming the position, the child was asked to straighten the lower leg. The O1 reset the inclinometer in a horizontal position, and then placed it below the lower aspect of the ischial tuberosity (Figure 3) and measured the popliteal angle.

### Modified finger-to-floor test (FTF) [37]

**Figure 4:**
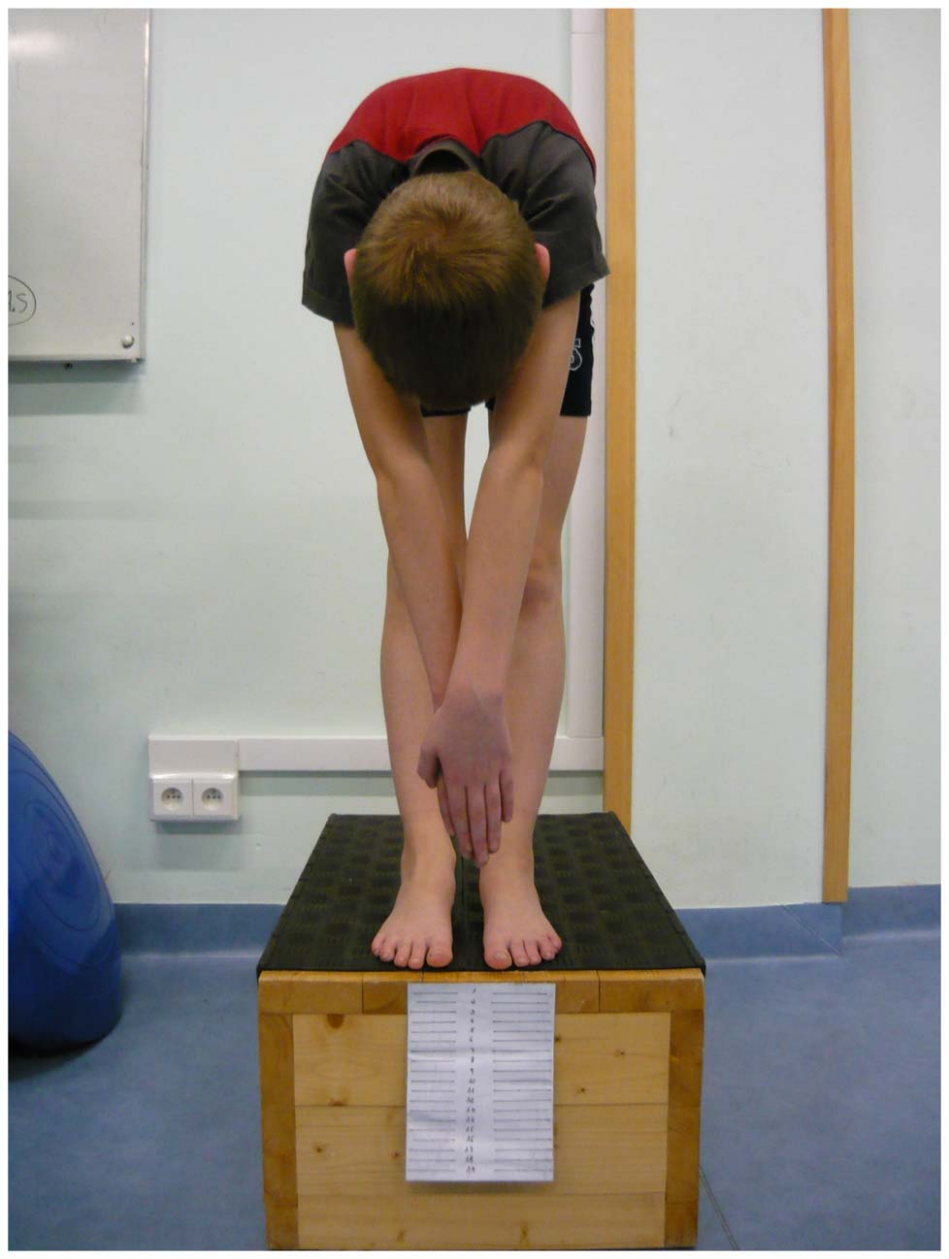
Finger-to-floor test.

The FTF was performed with the subject standing barefoot on a 30-cm-high measuring box. The subject bent forward and the distance from the fingertips to the top of the box was measured. Negative values (marked with a minus) denoted that the child was unable to reach the top of the measuring box, “zero” denoted the child was able to touch the box, positive values (no mark) denoted the child was able to touch the box below its top. The results were given in centimeters (Figure 4).

### Reliability study [39]

One week before the exercise program, the reliability of the measurements of the SLR, the PA and the FTF was assessed in 10 subjects. These were children randomly selected from the study group. Each measurement was taken three times and the mean value was used for the analysis. The measurements were repeated the following day.

### Subject randomization

After the initial examination, the children were randomly assigned in simple randomization procedures to one of the three groups: (1) the PIR group (n = 40), performing self-stretch by post-isometric relaxation, (2) the SS group (n = 40), performing static stretch and stabilizing exercises, and (3) the SE group (n = 40), performing stabilizing exercises (allocation ratio 1∶1∶1). In the randomization procedure each of the parents selected one envelope including a piece of paper with the number referring to one of the groups: PIR - 1; SS - 2,: SE - 3. The procedure was conducted by the main author of the study who did not participate in the measurement protocol and exercise program.

### Exercise program

Each group underwent a 6-week exercise program including one physiotherapy session per week and 6 sessions of daily home exercises per week. Two physiotherapists, both with minimum 2-year-long experience in children therapy, including PIR, static stretch and stabilizing managed the exercises. Before the exercise program the children were educated on how to attain and maintain the neutral position of the lumbo-pelvic-hip (LPH) complex [29], [40]. Each session with a physiotherapist started with a 5-minute exercise session aiming at increasing the ability to hold the neutral position of the LPH complex. The total set of exercises took about 10 minutes (5 minutes to control the LPH complex and 5 minutes for exercises). Each child received written instructions for home exercises which were identical to the ones performed with a physiotherapist. During the sessions with a physiotherapist, parents' presence was required so that they could observe and learn the exercises and control them at home.

### The PIR group exercise program

**Figure 5:**
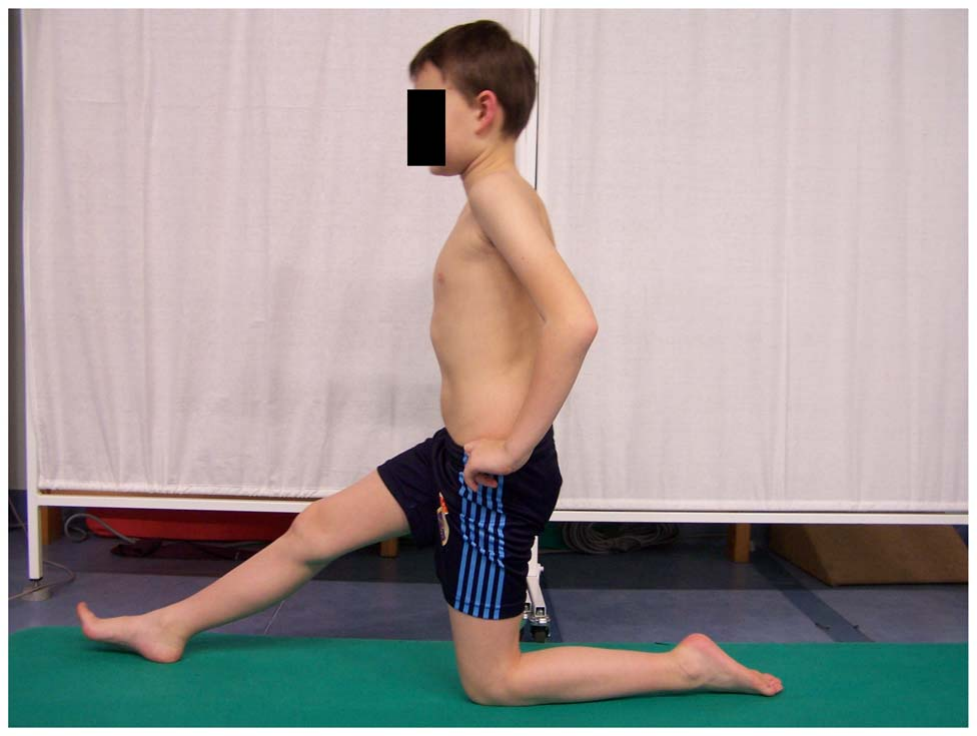
Post-isometric hamstring relaxation.

The program of post-isometric relaxation (the PIR group) was conducted according to Chaitow [21] and Lewit [22]. Kneeling on one knee was the start position [40] (Figure 5). The child performed anterior pelvic tilt with simultaneous trunk forward shift, without the loss of a neutral position of sagittal spinal curvatures, to the moment when a stretch in hamstring was felt. During the contraction phase the child was asked to slightly press the right heel to the floor. This phase lasted 10 seconds, and was followed by the relaxation phase with a gradual increase in the range of hip flexion. There were five sets of exercises with 10-second relaxation phases.

### The SS group exercise program

**Figure 6:**
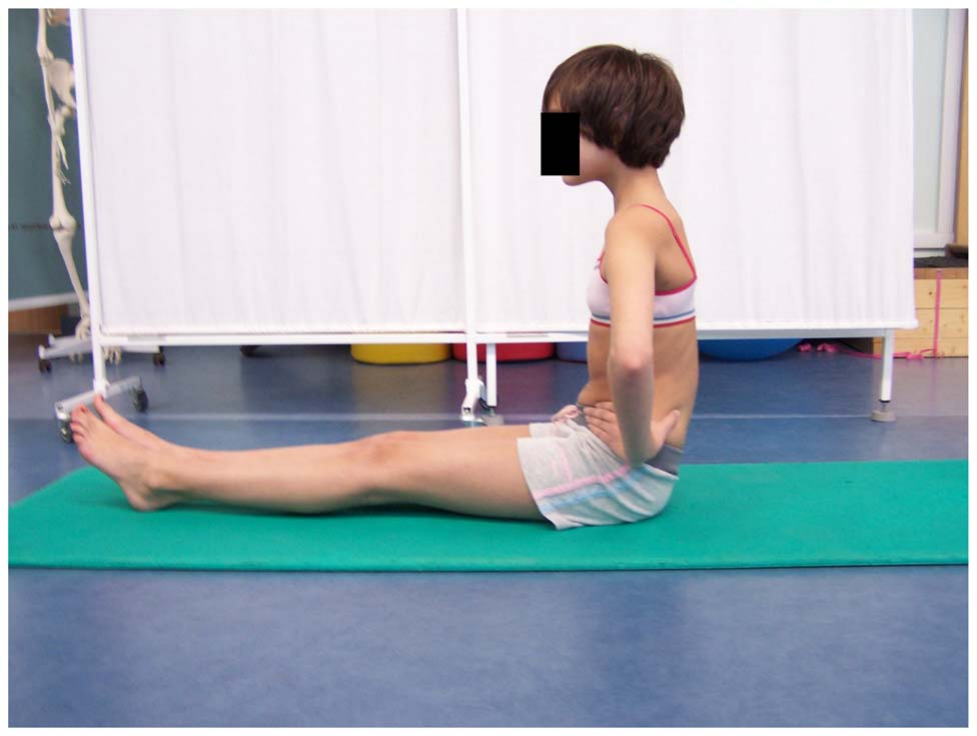
Static stretching of hamstrings.

**Figure 7:**
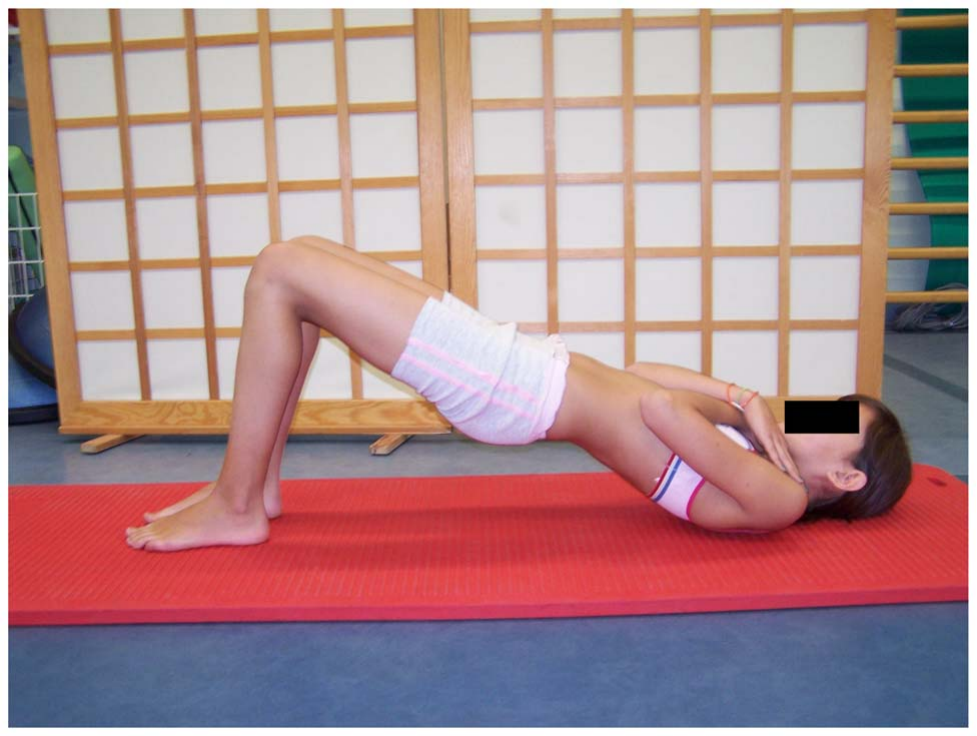
Gluteus maximus activation in a supine position.

**Figure 8:**
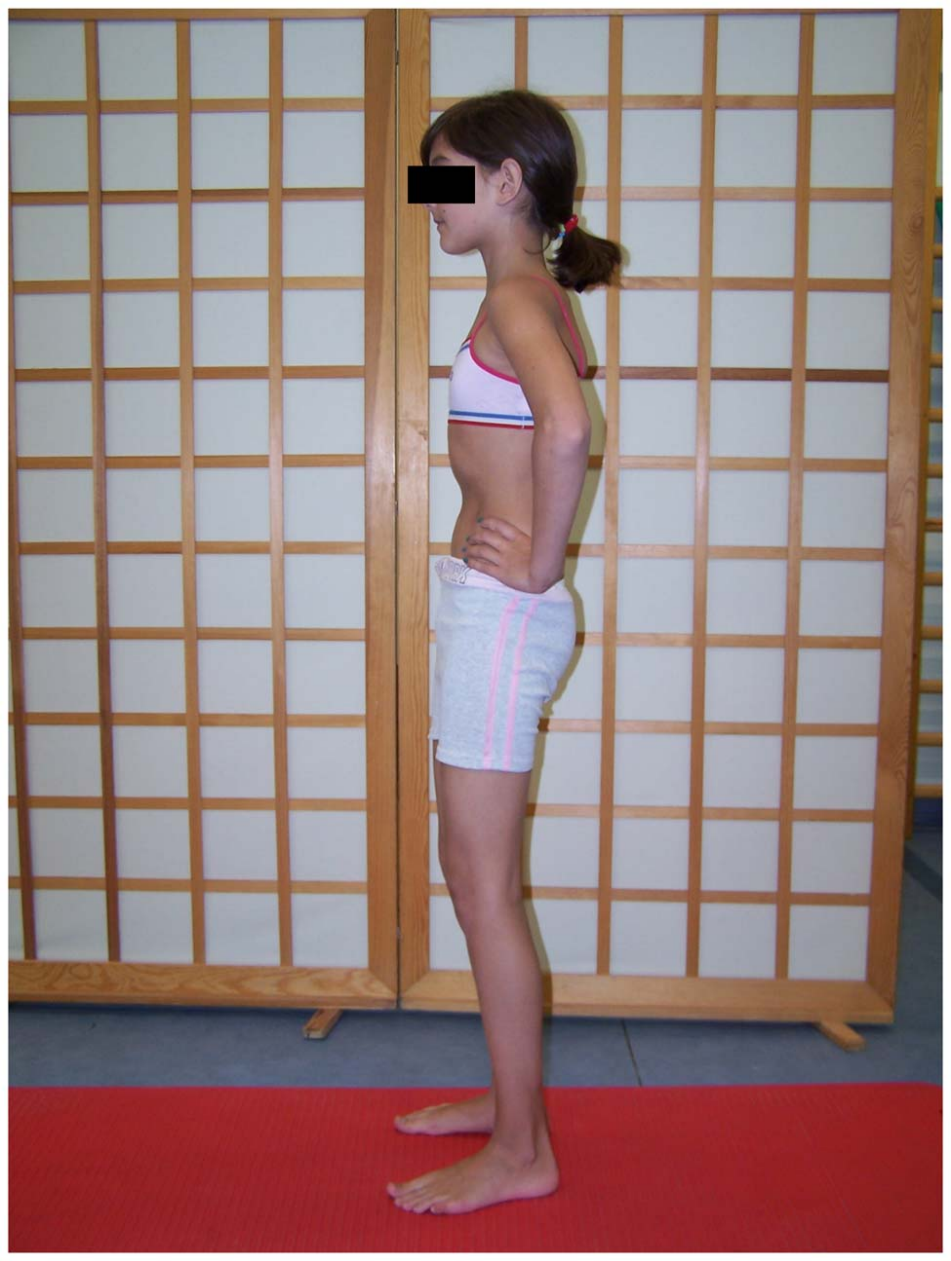
Gluteus maximus activation in a standing position.

The SS group performed static stretch for hamstring inhibition in a sitting position. In the first phase, the pelvis and the spine were in a free, kyphotic position, the knees extended, the feet relaxed. Then, the child bent the pelvis forward so as to feel the stretch in hamstring muscles. This position was held for 30 seconds and was followed by a 30-second break (Figure 6). The exercise was repeated four times. To activate the gluteus maximus, two exercises were performed. The first one was done with the subject in a supine position with hips and knees bent and feet supported on the ground. The child raised the pelvis to the level determined by the line joining knees and shoulders. This position with isometric activation was kept for 10 seconds and was followed by a 10-second break (Figure 7). The exercise was repeated 10 times. To avoid hamstring activation, the subjects were asked to activate their gluteus maximus and simultaneously press their feet against the ground forwards but without any visible movement. The second exercise was performed in a standing position with the hands on the pelvis. The child moved the posterior tilt of the pelvis at the same time activating the gluteus maximus (Figure 8) [27], [28], [40].

### The SE group exercise program

The SE group performed exercises aimed at activating gluteus maximus exclusively. These were the same exercises as the ones performed in the SS group. They are presented in Figure 7 and 8.

### Statistical analysis

Twenty-six children who did not participate in all the six exercise sessions with a physiotherapist were excluded from the statistical analysis (26 drop-outs). Therefore, the results obtained by 94 children were adopted to the analysis (32 children in the PIR group, 31 in the SS group, and 31 in the SE group) (Table 1).

**Table 1:** Parameters of the three groups at an initial examination after excluding data of drop-outs.

	PIR group n = 32	SS group n = 31	SE group n = 31	P level
	Mean (SD)	Mean (SD)	Mean (SD)	
Age (yrs)	11.4 (0.6)	11.5 (0.6)	11.5 (0.5)	0.84
Height (m)	1.5 (0.07)	1.5 (0.07)	1.5 (0.09)	0.83
Weight (kg)	43.5 (9.2)	45.4 (11.1)	43.3 (10.4)	0.67
BMI (kg m-2)	18.4 (3.2)	19.3 (3.6)	18.2 (3.4)	0.4
SLR (°)	47.4 (11.3)	48.3 (10.1)	44.7 (9.7)	0.053
PA (°)	52.3 (10.3)	53.3 (13.9)	50.2 (10.4)	0.55
FTF (cm)	−6.6 (7.5)	−5.7 (8.3)	−6.5 (7.9)	0.88

Abbreviations: PIR group – post-isometric relaxation group; SS group – static stretch with stabilizing exercises group; SE group – stabilizing exercises group; SLR test – straight leg raise test; PA – popliteal angle test; FTF – finger-to-floor distance in trunk flexion test.

The statistical analysis was made with Statistica 7.1 software (StatSoft, Poland). Normal distribution was assessed with the use of Shapiro-Wilk test. The ANOVA and Tukey tests were used to compare the results obtained in the three independent groups. Wilcoxon tests were applied to compare the results from dependent groups. To assess the reliability level, the Alpha Cronbach test was used [39]. The value P = 0.05 was adopted as the level of significance.

## Results

The reliability of measurements was as follows: 0.99 for the SLR, 0.99 for the PA, and 1.0 for the FTF. The measurement error was as follows: 2.9° for the SLR, 3.3° for the PA, and 1.5 cm for the FTF.

Before the exercise program there were no differences between the PIR, SS and SE groups regarding age, height, weight, BMI, SLR, PA, and FTF (Table 1).

During the final examination, a significant increase in all test results (SLR, PA, FTF) was observed both in the PIR and SS group. In the SE group, a significant increase was obtained in the SLR but not in the PA or the FTF (Table 2).

**Table 2:** Means, standard deviations (SD) and levels of significant differences in each group regarding the results obtained during the first and second examination.

		PIR group n = 32	SS group n = 31	SE group n = 31
		Mean (SD)	Mean (SD)	Mean (SD)
SLR (°)	Exam1	47.4 (11.3)	48.3 (10.1)	44.7 (9.7)
	Exam2	56.7 (11.0)†	59.4 (11.6)†	49.1 (10.7)†
PA (°)	Exam1	52.3 (10.3)	53.3 (13.9)	50.2 (10.4)
	Exam2	57.5 (11.4)**	61.0 (11.9)†	52.5 (11.7)
FTF (cm)	Exam1	−6.6 (7.5)	−5.7 (8.3)	−6.5 (7.9)
	Exam2	−3.1 (5.3)**	−2.7 (5.3)**	−5.4 (7.0)

**P<0.01;

† P<0.001.

Abbreviations: PIR group – post-isometric relaxation group; SS group – static stretch with stabilizing exercises group; SE group – stabilizing exercises group; SLR – straight leg raise test; PA – popliteal angle test; FTF – finger-to-floor distance in trunk flexion test.

During the final examination, the SLR results obtained in the PIR and SS groups, i.e. 56.7°±11.0, and 59.4°±11.6, respectively, were significantly higher than in the SE group: 49.1°±10.7. The SS group demonstrated a greater PA value than the SE group: 61.0°±11.9 vs. 52.5°±11.7. There was no significant difference in the PA test result between the PIR and SE group as well as between the PIR and SS group. There were no significant differences regarding the FTF results among the three groups (Table 3).

**Table 3:** Means, standard deviations (SD) and levels of significant differences regarding the results of SLR, PA and FTF tests obtained in all groups during the final examination.

	PIR group n = 32	SS group n = 31	SE group n = 31	P level	Tukey test
	Mean (SD)	Mean (SD)	Mean (SD)		
SLR (°)	56.7 (11.0)	59.4 (11.6)	49.1 (10.7)	<0.001	PIR>SESS>SE
PA (°)	57.5 (11.4)	61.0 (11.9)	52.5 (11.7)	0.014	SS>SE
FTF (cm)	−3.1 (5.3)	−2.7 (5.3)	−5.4 (7.0)	0.15	–

Abbreviations: PIR group – post-isometric relaxation group; SS group – static stretch with stabilizing exercises group; SE group – stabilizing exercises group; SLR – straight leg raise test; PA – popliteal angle test; FTF – finger-to-floor distance in trunk flexion test.

The biggest increase in the SLR was observed in the SS group (11.5°±7.4), but there were no significant differences regarding the results obtained in the PIR and SE groups. The biggest increase in the PA test was noted in the SS group (7.7°±8.5), but the difference between the groups was insignificant. There were no significant differences between all groups as far as the increase in the results of FTF is concerned (Table 4).

**Table 4:** Differences between Exam2 and Exam1 for all parameters.

	PIR group	SS group	SE group	P level
	Mean (SD)	Mean (SD)	Mean (SD)	
SLR (°)	9.3 (10.0)	11.5 (7.4)	4.4 (6.9)	0.14
PA (°)	5.2 (7.7)	7.7 (8.5)	2.3 (10.6)	0.06
FTF (cm)	3.5 (6.3)	2.9 (5.8)	1.0 (3.6)	0.15

Abbreviations: PIR group – post-isometric relaxation group; SS group – static stretch with stabilizing exercises group; SE group – stabilizing exercises group; SLR – straight leg raise test; PA – popliteal angle test; FTF – finger-to-floor distance in trunk flexion test.

## Discussion

The aim of this study was to evaluate the influence of post-isometric relaxation, static stretching combined with gluteus maximus activating exercises, and gluteus maximus strengthening exercises on hamstring flexibility. All the three methods are widely applied [21]–[25], [27]–[30], [40]; however, they have not been directly compared before.

The SLR, the PA and the FTF tests were used to assess hamstring flexibility [32]–[34], [37]. The evaluation of the reliability of measurements revealed a level higher than 0.9 for each of them. According to Bland and Altman [39], such a result proves excellent reliability. The level of measurement error also seems to be low, and therefore we believe that it did not influence the interpretation of the obtained results.

The stretch parameters chosen for this study were based on the data from the literature [21], [22], [27], [28], [40]. However, it is worth noting that optimal stretch parameters for stretching exercises are not established so it is difficult to make a direct comparison of results of various studies due to the variety of subjects' age, techniques applied, number of repetitions and sets, or duration of exercises.

The results obtained in this study indicate that after a 6-week exercise program the biggest improvement in hamstring flexibility was achieved by children performing post-isometric relaxation and static stretching combined with stabilizing exercises. Only in these groups a significant improvement in all the tests (SLR, PA, FTF) was achieved. However, it is difficult to decide which of these two methods is more efficient because the SLR result during the final examination was significantly higher both in the PIR and SS group than in the SE group. After the final examination the PA result was significantly higher only in the SS group compared to the SE group. However, there occurred no differences between the results obtained in the PIR and SS group in any case.

Feland et al. found that both post-isometric relaxation and static stretching are effective in increasing the SLR and did not find any difference regarding the effectiveness of both techniques [41]. A similar observation was made by Yuktasir and Kaya [42]. In turn, Magnusson et al. [23] found that in 10 males the implementation of an isometric contraction before the stretching phase led to a greater improvement in popliteal angle compared to passive stretching only. However, it is worth noting that Feland et al. [41], Yuktasir and Kaya [42] as well as Magnusson et al. [23], concentrated their studies on the adult populations and did not supplement static stretching with exercises aimed at gluteus maximus strengthening, as it was done in this study. Therefore, a caution is advised while directly comparing these results with the results of the present study.

As far as we know, the influence of exercises aiming at strengthening gluteus maximus on the change in hamstring flexibility in children has not been assessed yet. We find it interesting that exercises involving daily activation of gluteus maximus exclusively (SE group) resulted in a significant increase in the SLR result. This observation might support the theory of Mottram and Comerford [27] as well as Richardson et al. [43], [44] that increasing the activity of muscles responsible for stabilization (e.g. gluteus maximus) leads to an inhibition of global muscles (e.g. hamstring muscles). Wagner et al. confirmed that the training concentrating on gluteus maximus strength leads to an increase in hamstring length [30]. However, these authors analyzed adult males with exercise-associated muscle cramping [30].

On the other hand, in the present study no positive influence of these exercises was noted in the PA and the FTF tests. The presumed reason might be the fact that in the PIR and SS groups exercises may have increased the flexibility of other muscles (e.g. gastrocnemius), which might have influenced the results of the PA and the FTF. Another reason might be that the stabilizing exercises may not be as effective in increasing the flexibility of muscles as the exercises including stretching techniques. In our opinion, the results of the present study, which indicate that limiting to stabilizing exercises may be to a certain extent effective in increasing hamstring flexibility, could be the basis for carrying out further studies evaluating the influence of various types of stabilizing exercises on hamstring and other muscles flexibility.

Although the observers used the same instructions and guidelines, some children needed more instructions and assistance to perform exercises correctly. We believe that it did not influence the results significantly because the likelihood that children demanding more assistance were present in a given group was the same for all groups. A similar opinion was presented by Schuback et al. [45]. For the same reason, different levels of engagement in performing home exercises might also prove insignificant.

## Conclusions

A 6-week training of post-isometric muscle relaxation and static stretch with stabilizing exercises exerted a similar influence on increasing hamstring flexibility and trunk forward bend in healthy 10- to 13-year-old children, as expressed by the improvement in the straight leg raise, popliteal angle and finger-to-floor test results.

The exercises limited to the straightening of gluteus maximus resulted in a significant increase in the SLR test result, but not PA or FTF test results.
